# Genome-Wide Dissection of the Dof Transcription Factor Family in *Senna tora*: From Gene Identification to Expression Signatures

**DOI:** 10.3390/biology15201832

**Published:** 2026-10-13

**Authors:** Shuang Liu, Yili Zhang, Jiayao Lyu

**Affiliations:** Shaanxi Province Key Laboratory of Bio-Resources, Qinba Mountain Area Collaborative Innovation Center of Bioresources Comprehensive Development, Qinba State Key Laboratory of Biological Resources and Ecological Environment, School of Biological Sciences and Engineering, Shaanxi University of Technology, Hanzhong 723001, China; 19145339807@163.com (Y.Z.);

**Keywords:** *Dof* transcription factor, *Senna tora*, genome-wide identification, phylogenetic analysis, expression profiling

## Abstract

*Dof* (DNA binding with one finger) transcription factors constitute a plant-specific family of transcriptional regulators that play extensive roles in plant growth, development, and secondary metabolism. However, a systematic identification and evolutionary analysis of the *Dof* gene family in the medicinal plant *Senna tora* (*S. tora*) has not yet been reported. In this study, a genome-wide identification and phylogenetic analysis of the *Dof* gene family in *S. tora* was performed to elucidate its evolutionary patterns and potential functions. The results revealed a total of 36 *StDof* genes in the *S. tora* genome, which were classified into four subfamilies, with subgroup D representing the largest proportion. Collinearity analysis further revealed the evolutionary history of the *StDof* gene family, identifying 23 gene pairs, with Ka/Ks values for all homologous gene pairs being less than 1. It was also found that *StDof* promoters contain a large number of regulatory elements associated with hormone responses, light signaling, and various abiotic stresses. Furthermore, the expression profiles of *StDof* genes at seven different developmental stages of the seed demonstrated that 18 genes were preferentially expressed in early development, whereas 18 genes were preferentially expressed in late development. The differentially expressed genes, *StDof9* (upregulated) and *StDof16* (downregulated), were successfully identified. Subcellular localization experiments confirmed that the candidate genes *StDof9* and *StDof16* exert their biological functions within the nucleus, consistent with the prediction results. Taken together, this study provides a theoretical foundation for understanding the evolutionary history of the *Dof* gene family in *S. tora* and lays the groundwork for future functional characterization of *StDof* genes.

## Simple Summary

*Dof* transcription factors constitute a plant-specific gene family governing growth, development, and secondary metabolism. Here, we present the first genome-wide characterization of the *Dof* family in the medicinal legume *Senna tora*. A total of 36 *StDof* genes were identified and phylogenetically classified into four subfamilies. Evolutionary analysis revealed that segmental duplications drove family expansion, with all orthologous pairs undergoing purifying selection (Ka/Ks < 1). Promoter analysis indicated enrichment for cis-elements responsive to phytohormones, light, and abiotic stress. Spatiotemporal expression profiling across seven seed developmental stages delineated two distinct clusters: 18 genes peaking in early development and 18 in late maturation. Crucially, the differentially expressed *StDof9* and *StDof16* were validated as nuclear-localized proteins. This study systematically analyzed the expansion mechanisms and expression characteristics of the *StDof* gene family, providing an important basis for the in-depth identification of candidate functional genes.

## 1. Introduction

Transcriptional regulation is a core molecular mechanism underlying plant life cycle progression and adaptive environmental reprogramming. Among the numerous plant-specific transcription factor families, the *Dof* (DNA binding with One Finger) family has emerged as a research hotspot owing to its unique C2-C2-type single zinc finger structure and high species specificity. The N-terminal region of Dof proteins contains a conserved zinc finger domain of approximately 50 amino acids that specifically recognizes and binds to the AAAG/CTTT core motif in the promoter regions of target genes, thereby mediating transcriptional activation or repression [1,2]. Since the initial cloning of the *Dof* gene from maize in 1993, numerous studies have demonstrated that members of the *Dof* family play a significant role in regulating various biological processes [3], including phytohormone signal transduction, photoperiod response, carbon and nitrogen metabolism, organ development, and abiotic and biotic stress responses [4].

In recent years, with the rapid development of high-throughput sequencing and comparative genomics, genome-wide identification of the *Dof* family has been systematically completed in model and crop species such as *Arabidopsis thaliana* (*A. thaliana*), rice, soybean, maize, wheat, potato, *Morus notabilis* (*M. notabilis*), etc. [5,6,7,8,9,10,11]. Functional validation studies have further revealed the core regulatory roles of Dof proteins from multiple dimensions. In terms of stress and hormone responses, the *atcdf3-1* loss-of-function mutant is highly sensitive to drought and cold stress, whereas *AtCDF3* overexpression lines significantly enhance stress tolerance by improving osmotic adjustment capacity [12]. The overexpression of *AtCDF4* enhances the biosynthesis of abscisic acid (ABA) and the accumulation of reactive oxygen species (ROS), thereby expediting leaf senescence and the abscission of floral organs in *A. thaliana* [13]. In seed development and carbon metabolism regulation, knockout of *ZmDof3* in maize reduces the expression of starch synthesis genes and starch accumulation in seeds [14]. Conversely, the overexpression of *ZmDof36* enhances the activation of *ZmAGPS1a* and *ZmGBSSI*, thereby promoting increased starch accumulation [15]. In kiwifruit, *AdDof3* facilitates fruit ripening and softening by activating *AdBAM3L*, which influences flavor development [16]. In terms of secondary metabolism and quality regulation, *CsDof1.8* has been demonstrated to influence the biosynthesis of C9 aldehydes and their corresponding alcohols, thereby directly affecting the accumulation of characteristic fruit aroma volatiles in cucumber [17]. In lipid metabolism, *GmDof4* and *GmDof11* enhance soybean seed oil content by upregulating genes in the fatty acid biosynthesis pathway [18]. Similarly, the overexpression of *GmDof4* in *Chlorella ellipsoidea* (*C. ellipsoidea*) resulted in a 50% increase in lipid content compared to the wild type [19]. Silencing of *BnDof5.6* shifts the fatty acid composition of rapeseed from long-chain fatty acids toward saturated fatty acids [20]. In summary, cross-species evidence unequivocally indicates that *Dof* transcription factors serve as central regulatory hubs in essential plant growth and development. They are also critical targets for enhancing crop quality and breeding for stress tolerance. Nevertheless, research has predominantly concentrated on model plants and major grain and oil crops. In contrast, specialty crops such as *Senna tora* (*S. tora*), which are valued for both medicinal bioactive compound accumulation and oilseed potential, have not been thoroughly investigated. Specifically, the genome-wide characteristics, evolutionary patterns, and functional roles of the *Dof* gene family in seed development and stress adaptation in *S. tora* require systematic elucidation.

*S. tora* is an annual subshrubby leguminous herb, primarily utilized for its medicinal seeds [21]. Meanwhile, *S. tora* also serves as an important green manure crop and cover plant in tropical and subtropical regions, possessing considerable application value in the integrated system of medicine–food homology industries and agroecological agriculture. In recent years, a series of studies have been conducted on the evaluation of *S. tora* germplasm resources and the breeding of new varieties, including the screening of *S. tora* lines with superior agronomic traits and high anthraquinone content through mutagenesis breeding [22,23,24], as well as the elucidation of the molecular mechanisms by which *StTCP11* and *StDA1* regulate anthraquinone biosynthesis [25,26]. However, existing breeding achievements remain insufficient to fully address the fluctuations in the yield and quality of *S. tora* under complex cultivation environments, and the underlying molecular regulatory networks still require in-depth dissection. *Dof* transcription factors, which belong to the plant-specific zinc finger protein superfamily, play a significant role in various physiological processes, including seed development, secondary metabolite accumulation, and responses to abiotic stress. To date, genome-wide identification and functional characterization of the *Dof* transcription factor family in *S. tora* remain unexplored. Therefore, a systematic study of the structural characteristics and regulatory mechanisms of the *StDof* gene family is of great significance for elucidating the molecular basis of important traits in *S. tora* and for advancing the field of molecular breeding.

In this study, a total of 36 *StDof* genes were identified and classified into four subfamilies in *S. tora*. A comprehensive analysis of the *StDof* genes was conducted using bioinformatics methods to examine their motif composition, cis-acting regulatory elements in the promoter region, gene structure, phylogenetic relationships, chromosomal localization, and syntenic associations. Concurrently, the expression patterns of *StDofs* at different stages of seed development were investigated. Furthermore, the subcellular localization of *StDof9* and *StDof16* was determined through transient expression experiments in tobacco leaves. The findings of this study provide an important foundation for future functional characterization of the *Dof* gene family in *S. tora*.

## 2. Materials and Methods

### 2.1. Search and Identification of StDofs

To systematically identify members of the *Dof* transcription factor family in *S. tora*, this study employed three strategies: homology-based alignment, HMMER search, and domain validation. First, full-length protein sequences of the known *Dof* gene family in *A. thaliana* were downloaded from the TAIR database (https://www.arabidopsis.org/) as a reference, and a homology search against the predicted proteome of *S. tora* was performed using the BLASTp tool (E-value < 1 × 10^−14^) (https://blast.ncbi.nlm.nih.gov/Blast.cgi, accessed on 25 June 2026). Concurrently, the hidden Markov model (HMM) of the canonical Dof conserved domain (PF02701) was retrieved from the Pfam database, and a genome-wide search was conducted using HMMER (v 3.3.2), based on this model. Candidate sequences obtained from both approaches were submitted to the NCBI CDD (https://www.ncbi.nlm.nih.gov/cdd, accessed on 26 June 2026) and the Pfam database (https://www.ebi.ac.uk/interpro/entry/pfam/, accessed on 26 June 2026) for dual validation of conserved domains. Entries lacking the complete canonical Dof domain or exhibiting sequence redundancy were removed, thereby yielding the final set of *StDof* gene family members.

### 2.2. Physicochemical Property Analysis of the StDofs

The molecular weight (MW) and isoelectric point (pI) of all identified StDof protein sequences were predicted using the ExPASy software (https://www.expasy.org/, accessed on 30 June 2026). Subcellular localization was analyzed using the online tool Cell PLoc (http://www.csbio.sjtu.edu.cn/bioinf/Cell-PLoc/, accessed on 3 July 2026). The secondary structural composition of all identified StDof proteins, including the fractional content of α-helices, extended strands, β-turns, and random coils, was predicted using the Self-Optimized Prediction Method with Alignment (SOPMA) implemented in the Network Protein Sequence Analysis (NPSA) platform (https://npsa.lyon.inserm.fr/cgi-bin/npsa_automat.pl?page=/NPSA/npsa_sopma.html, accessed on 3 July 2026). Full-length tertiary structures were subsequently modeled via the Phyre2.2 homology recognition server (http://www.sbg.bio.ic.ac.uk/phyre2/html/page.cgi?id=index, accessed on 6 July 2026), with all resulting structural models visualized and rendered using PyMOL (v3.0.3).

### 2.3. StDofs Chromosomal Location and Synteny Analysis

Based on the whole-genome annotation file of *S. tora*, the chromosomal localization information of *StDof* gene family members was extracted using the Gene Location Visualize module of TBtools (v2.487) [27,28]. Subsequently, intraspecific and interspecific collinearity analyses were performed between *S. tora* and *A. thaliana*, as well as between *S. tora* and rice, using the built-in McScanX plugin of TBtools (v2.487). For all identified homologous *StDof* gene pairs, the nonsynonymous substitution rate (Ka), synonymous substitution rate (Ks), and Ka/Ks ratio were calculated using the Simple Ka/Ks Calculator.

### 2.4. Phylogenetic Analysis of the Dof Proteins

To explore the phylogenetic relationships of StDof proteins in *S. tora*, a phylogenetic tree was constructed using MEGA X (v10.2.6) based on the inferred StDof protein sequences and Dof protein sequences from rice and *A. thaliana*. The optimal amino acid substitution model was automatically selected. Moreover, the bootstrap value was set to 1000, with all other parameters left at their default settings, and the tree was visualized using iTOL (https://itol.embl.de/, accessed on 17 September 2026).

### 2.5. Conserved Domain and Gene Structure Analysis of StDof Genes

The MEGA X software (v10.2.6) was used to align the conserved domain sequences of all StDof proteins. The conserved motifs of StDof proteins were analyzed using the MEME online tool (https://meme-suite.org/meme/tools/meme, accessed on 18 September 2026), with the maximum number of motifs set to 10 and all other parameters set to default. The conserved domain annotation information of StDof proteins was retrieved from the NCBI online database. The CDSs and other basic data of the *StDof* genes were batch-extracted from the whole-genome annotation file of *S. tora* using the TBtools software.

### 2.6. Cis-Acting Element Analysis of StDof Genes

The 2.0 kb upstream promoter sequences of all *StDofs* were extracted using TBtools and submitted to the PlantCARE online database (http://bioinformatics.psb.ugent.be/webtools/plantcare/html/, accessed on 19 September 2026) to predict cis-acting regulatory elements. Based on the prediction results, TBtools was subsequently used to generate linear distribution maps of the promoter cis-elements and gene expression heatmaps.

### 2.7. Prediction of Protein–Protein Interaction and miRNA Target Genes

To predict potential interaction partners of StDof proteins, the StDof amino acid sequences were aligned against the *A. thaliana* protein database to identify the corresponding Arabidopsis orthologs. The potential interactions among the aforementioned homologous proteins were identified based on the STRING database.

Additionally, mature miRNA sequences from soybean were obtained from the PmiREN3.0 database (https://pmiren.com/search, accessed on 20 September 2026 and submitted together with the CDSs of StDof family members to psRNATarget (https://www.zhaolab.org/psRNATarget/, accessed on 22 September 2026) for target prediction. High-confidence miRNA–StDof pairs were retained at an expectation threshold of ≤3.0, and the miRNA–StDof interaction network was visualized using the advanced enrichment c-net network tool on the Metware Cloud platform.

### 2.8. StDof Expression Pattern Analysis

In this study, transcriptome data from seven stages of seed development were retrieved from the NCBI SRA database (http://www.ncbi.nlm.nih.gov/sra, accessed on 12 July 2026) with the corresponding accession numbers SRX7700171-SRX7700176 and SRX7700182-SRX7700189. Each developmental stage contained two biological replicates. The *S. tora* transcriptome was assembled and filtered according to the method described by Liu et al. [29]. All transcriptome analyses were performed based on the *S. tora* reference genome ASM1485142v1. Raw RNA-seq reads were filtered for quality control to remove adapter sequences and low-quality reads. Clean reads were mapped to the reference genome using HISAT2, and gene expression levels were calculated as FPKM values. Expression profile heatmaps were constructed based on the log2(FPKM+1) values, which were normalized row-wise using Z-score transformation prior to visualization. Advanced clustering heatmaps were generated to display gene expression patterns. In addition, correlations between samples and between genes were calculated and visualized by correlation clustering heatmaps using the Metware Cloud tool (https://www.omicstudio.cn/tool?order=complex, accessed on 25 September 2026). Differential expression analysis was performed using DESeq2, and the Benjamini–Hochberg method was used to correct *p*-values for multiple testing. The visualization of differentially expressed genes was performed on Metware Cloud by generating fold-change volcano plots. All parameters mentioned above were set to default values.

### 2.9. Subcellular Localization of StDofs

The coding sequences (CDS) of *StDof9* and *StDof16* were cloned into the pCambia1300-GFP vector using the ClonExpress Ultra One Step Cloning Kit V2 (Vazyme, Nanjing, China), yielding 35S::StDof9-GFP and 35S::StDof16-GFP, respectively. *StTCP4.1*, an experimentally verified nuclear-localized transcription factor of *S. tora* from our previous study [29], was used solely as a positional reference to mark the location of cell nuclei, rather than a Dof-family-specific control. Therefore, *StTCP4.1* was cloned into the pCambia1300-mCherry vector to serve as a control.

*Nicotiana benthamiana* (*N. benthamiana*) were cultivated in a growth chamber maintained at 25 °C under a 16 h light/8 h dark photoperiod with 60–70% relative humidity. Leaves (3–5 cm in length) from 4- to 5-week-old plants were selected for agroinfiltration. Equal amounts of Agrobacterium carrying 35S::StDof9-GFP, 35S::StDof16-GFP and 35S::StTCP4.1-mCherry constructs were mixed and co-infiltrated into tobacco leaves. The results were observed after 3–4 days. The primers used are listed in Table S1.

## 3. Results

### 3.1. The Identification of the Dof Gene Family in S. tora

The *Dof* gene family is a plant-specific family of transcription factors that exerts a significant influence on stress responses, biosynthesis of secondary metabolites, and plant growth and development. A total of 36 *StDof* genes were characterized in *S. tora* using BLASTP searches in combination with HMMER models. At the same time, based on their order of arrangement on the chromosomes, they have been named *StDof1* through *StDof36*. The structural composition and physicochemical properties of 36 *Dof* genes were analyzed in depth using the ExPASy software. The results showed that the amino acid lengths encoded by StDofs ranged from 159 aa (StDof34) to 521aa (StDof17), with molecular weights (MW) ranging from 18.07 kDa (StDof34) to 57.65 kDa (StDof17). The theoretical isoelectric points (pI) of these proteins ranged from 5.73 (StDof19) to 9.53 (StDof27). Among them, StDof5, StDof7, StDof9, StDof13, StDof15, StDof19, StDof25, StDof29, StDof31, StDof32, and StDof33 exhibited pI values below 7.0 (11 in total), whereas the remaining 25 StDof proteins had pI values above 7.0, indicating that the StDof protein family contains a relatively high proportion of basic amino acids. All StDof members of *S. tora* had negative grand average of hydropathicity (GRAVY) scores, indicating that they are hydrophilic proteins. Subcellular localization prediction results revealed that *StDof4* was localized to the plasma membrane, *StDof3/6/34* were localized to the chloroplast, *StDof25* was localized to the cell wall, and *StDof8* was localized to the mitochondria, while the remaining *StDofs* were all localized to the nucleus, suggesting that this gene family plays a potentially important role in the nucleus (Table 1).

**Figure 1:**
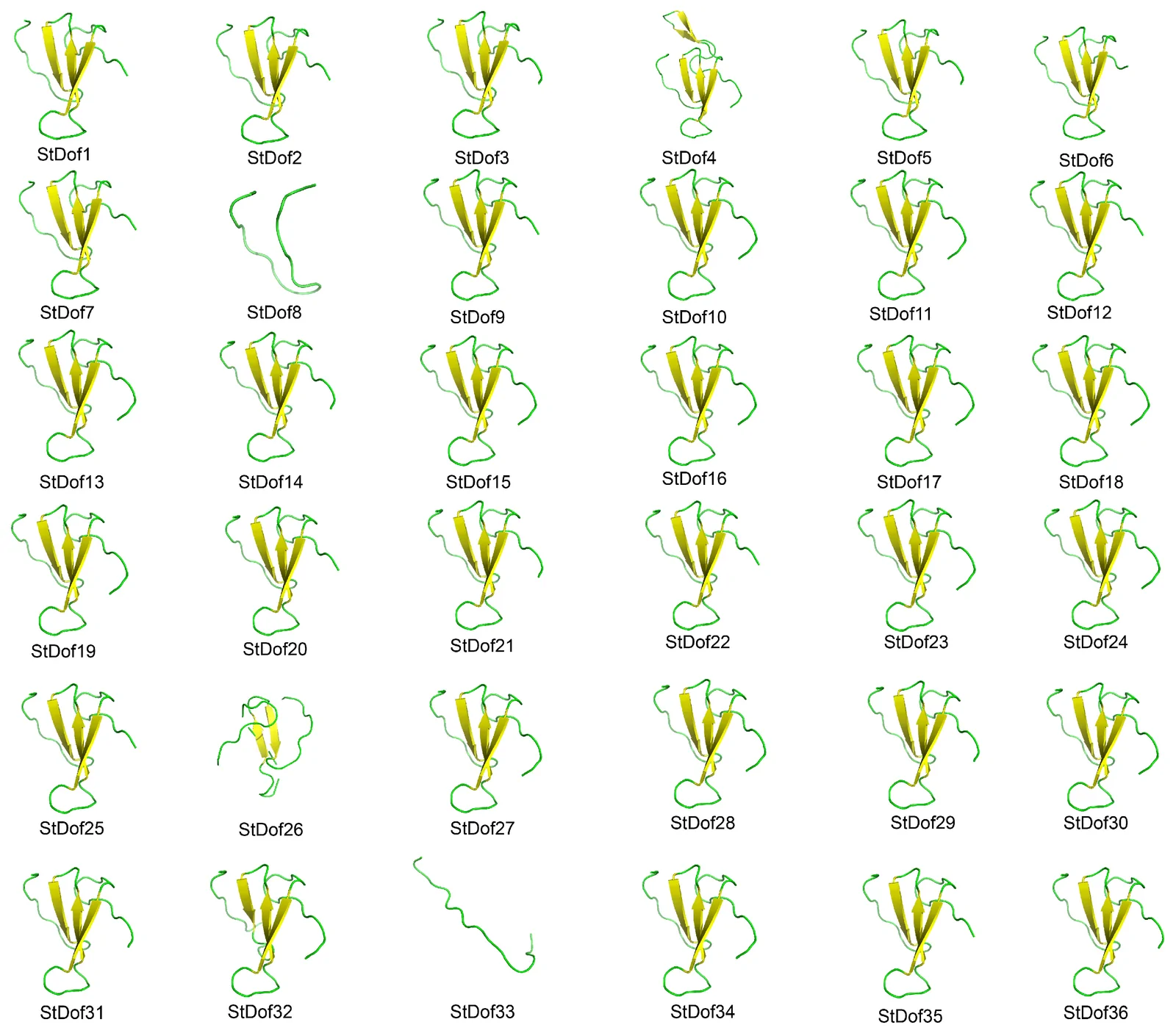
Tertiary structure of Dof proteins from *S. tora*.

Moreover, the secondary structure prediction results (Table S2) indicated that all members of the *StDof* gene family consist of α-helices, extended strands, β-turns, and random coils. Among these, disordered coils accounted for the highest proportion, ranging from 50.74% to 92.17%, while β-turns accounted for the lowest proportion, ranging from 0.48% to 4.93%. The predicted tertiary structures of these proteins also exhibit similar structural characteristics (Figure 1).

### 3.2. Chromosomal Localization of the StDof Gene Family

**Figure 2:**
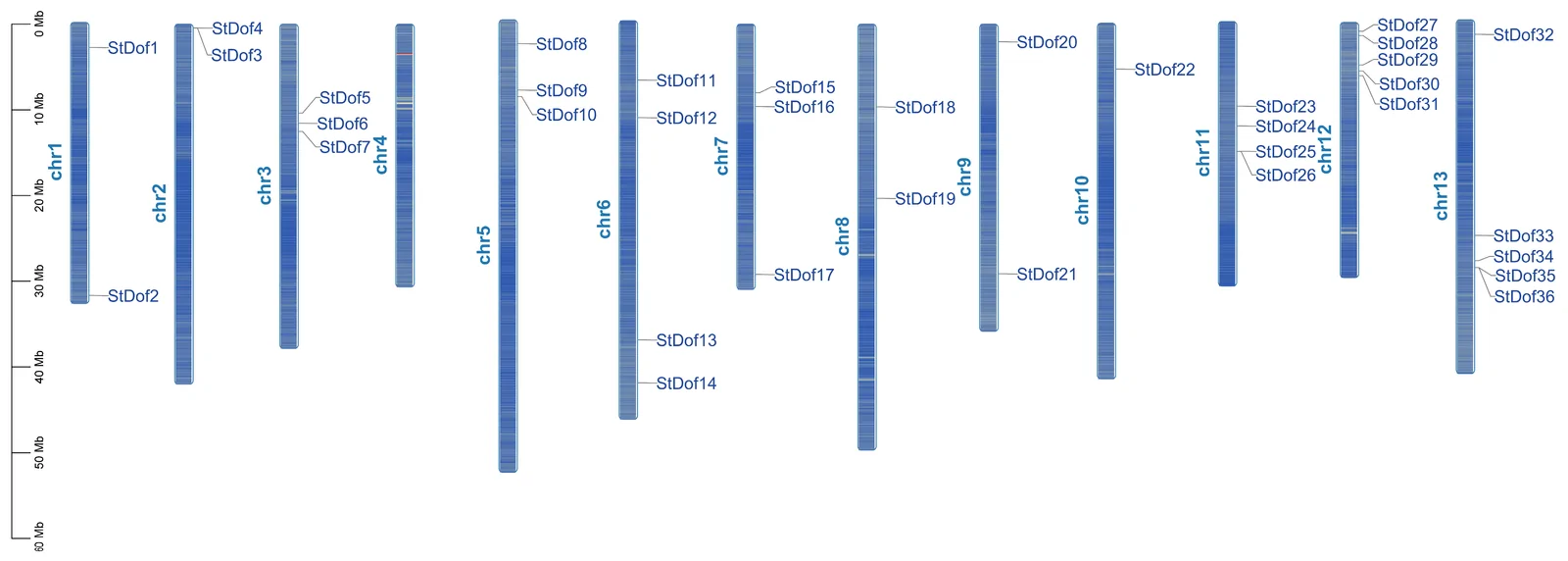
Chromosomal location of the *StDof* gene family. The vertical bars represent the chromosomes (chr1–chr13). The internal scale lines and gradient background constitute a gene density heatmap, where color variations indicate the gene density of the corresponding genomic regions (red indicates high density, while blue indicates low density). Labeled leader lines point to the specific physical locations of *StDof* gene family members on the chromosomes.

To elucidate the chromosomal localization patterns of *Dof* transcription factors in *S. tora*, a statistical analysis was conducted on the chromosomal distribution of the 36 putative *StDof* members. The results revealed that the *StDof* genes exhibited a markedly non-uniform distribution across 13 chromosomes, with chromosomes 13 and 12 harboring the largest number of family members (five in total), whereas chromosome 10 contained only a single member, representing the chromosome with the fewest *StDof* members. The remaining chromosomes each carried two or more *StDof* genes (Figure 2). Overall, the *Dof* gene family members were unevenly distributed across chromosomes, displaying distinct clustering in specific chromosomal regions. This nonrandom localization pattern partially reflects the expansion trajectory and potential for functional diversification during genomic evolution.

### 3.3. Collinearity Analysis of the StDof Gene Family

**Figure 3:**
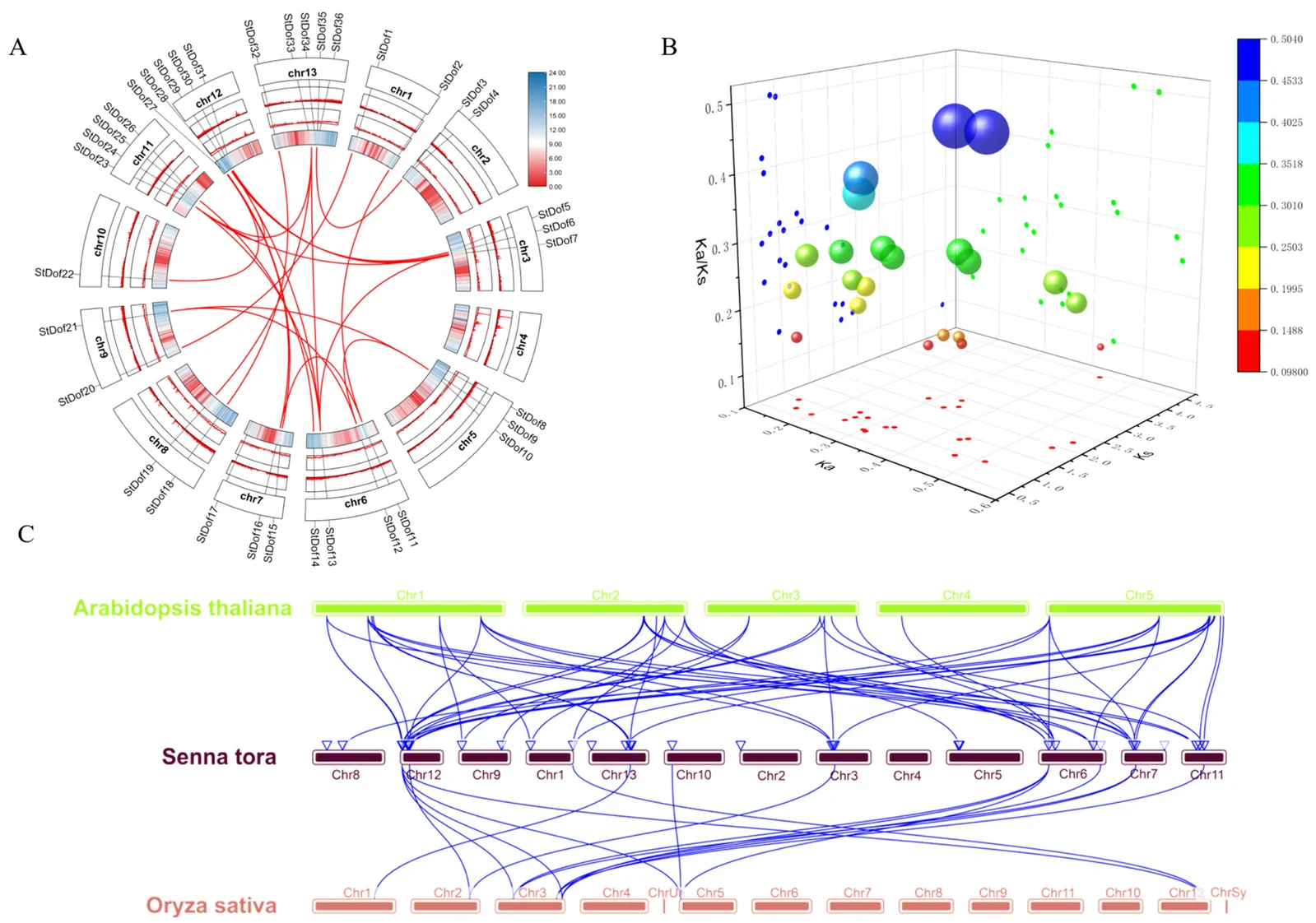
Collinearity and evolutionary selection pressure analysis of the *StDof* gene family. (**A**) Collinearity analysis of the *Dof* gene in *S. tora*; (**B**) analysis of evolutionary selection pressure (Ka/Ks) for *Dof* gene pairs. Three-dimensional plot: *X*-axis, Ka; *Z*-axis, Ks; *Y*-axis, and Ka/Ks. Colored spheres represent gene pairs; (**C**) collinearity analysis of *Dof* genes between *S. tora* and *A. thaliana*/rice.

To elucidate the intraspecific evolutionary characteristics of the *StDof* family in *S. tora*, a total of 23 collinear *StDof* gene pairs were identified (Figure 3A). Subsequently, the synonymous substitution rate (Ks), nonsynonymous substitution rate (Ka), and Ka/Ks ratio were calculated for all collinear gene pairs. The results indicated that the Ka/Ks values for all gene pairs were less than 1, with an overall range of 0 to 0.51 (Figure 3B), reflecting that this gene family is primarily subject to purifying selection (Ka/Ks < 1, =1, and >1 correspond to purifying selection, neutral evolution, and positive selection, respectively). Ks values predominantly ranged from 0.6 to 4.5 (Table S3), showing a broad distribution of Ks values among collinear gene pairs. Simultaneously, the Ka/Ks ratio for all gene pairs was less than 1, suggesting no signals of positive selection. Thus, purifying selection is the primary evolutionary driver that ensures the structural and functional conservation of *StDof* genes.

To elucidate the phylogenetic origin and evolutionary patterns of the *StDof* family in *S. tora*, comparative collinearity maps were constructed between the *Dof* gene families of *S. tora*, rice, and *A. thaliana* (Figure 3C). The finding revealed significant systematic evolutionary associations between the *StDof* and the *Dof* gene families of other species. Specifically, 16 and 54 orthologous gene pairs (including one-to-many gene relationships) were identified between the *Dof* genes of *S. tora* and those of rice (monocot) and *A. thaliana* (dicot), respectively. Among these, *StDof27* and *StDof30* exhibited five orthologous relationships with *A. thaliana Dof* genes, respectively. *StDof27* displayed four orthologous relationships with rice *Dof* genes. Notably, certain *StDof* genes showed collinearity exclusively with *A. thaliana Dof* genes, with no corresponding collinear relationships detected in rice. This distribution pattern not only confirms that *S. tora*, as a dicotyledonous plant, shares a closer common ancestry with *A. thaliana*, but also reveals that the *Dof* gene family underwent independent evolutionary and functional diversification within each lineage following the divergence between monocots and dicots. Taken together, the cross-species collinearity analysis provides crucial genomic evidence for elucidating the functional conservation and diversification mechanisms of the *Dof* family.

### 3.4. Phylogenetic Analysis of StDof Gene Family

**Figure 4:**
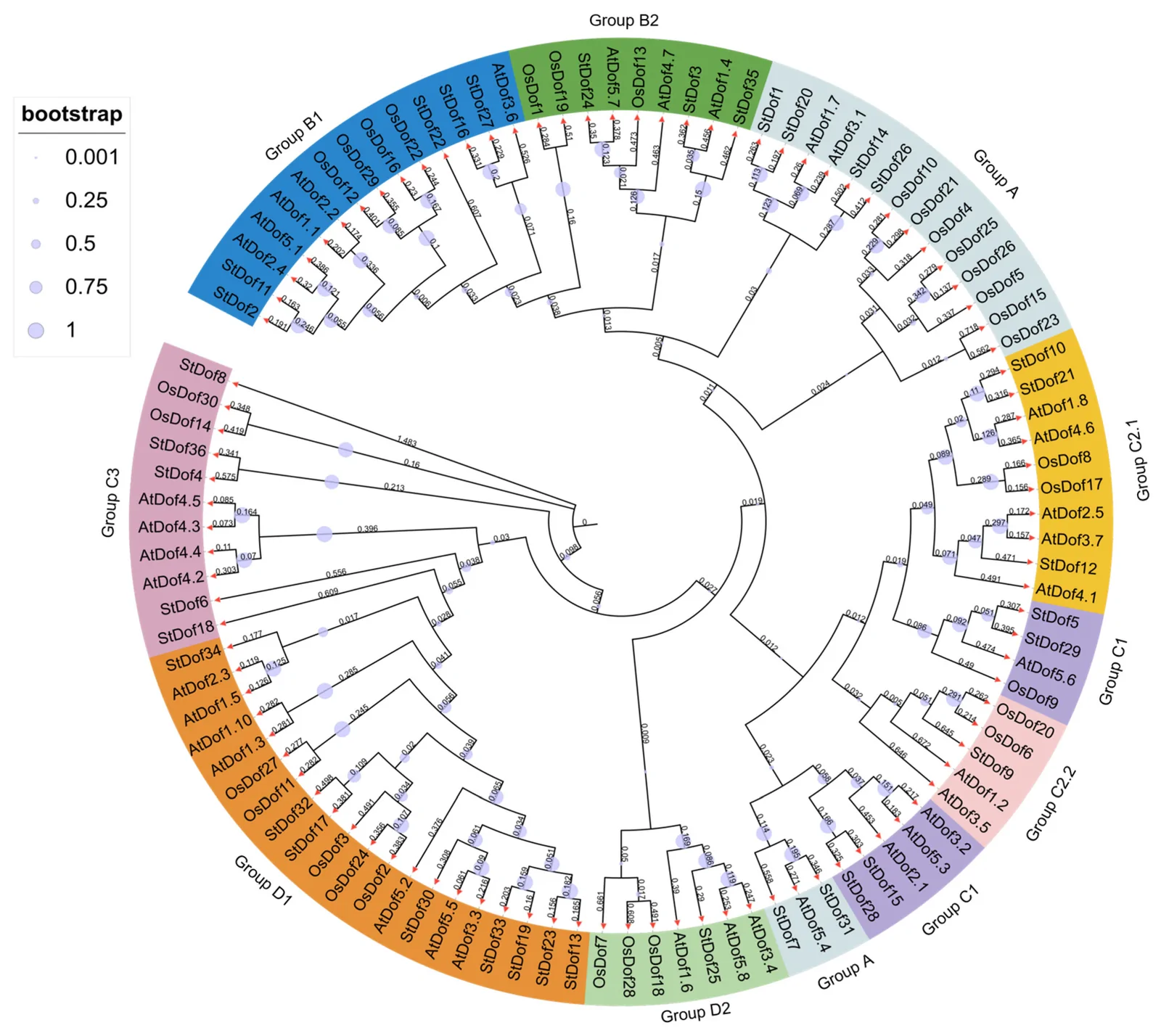
Phylogenetic analysis of the Dof protein family in *S. tora* and other species.

To elucidate the phylogenetic relationships and evolutionary characteristics of *Dof* transcription factors in *S. tora*, multiple sequence alignment of full-length Dof protein sequences from *S. tora*, rice, and *A. thaliana* was performed using the ClustalW program in MEGA X software (v10.2.6). Based on the alignment results, a phylogenetic tree was constructed using the neighbor-joining (NJ) method. Following the established classification criteria for AtDof proteins, the 36 putative StDof proteins were ultimately classified into four subfamilies (A, B, C, and D) and nine subgroups (A, B1, B2, C1, C2.1, C2.2, C3, D1, and D2). In terms of quantitative distribution, subgroup A contained only six StDof members, representing the lowest proportion, whereas subgroup C comprised 13 members, accounting for the highest proportion (Figure 4). Further sequence and domain analyses revealed that all StDof members harbored a highly conserved C2-C2-type zinc-finger DNA-binding domain (Figure S1), a hallmark feature of the Dof protein family, and that members within the same subgroup exhibited greater similarity in motif composition and sequence characteristics. These results not only clarify the evolutionary relationships of the StDof protein family but also suggest that StDof members within the same subgroup may possess conserved potential biological functions.

### 3.5. StDof Gene Structure and Conserved Motif Analysis

**Figure 5:**
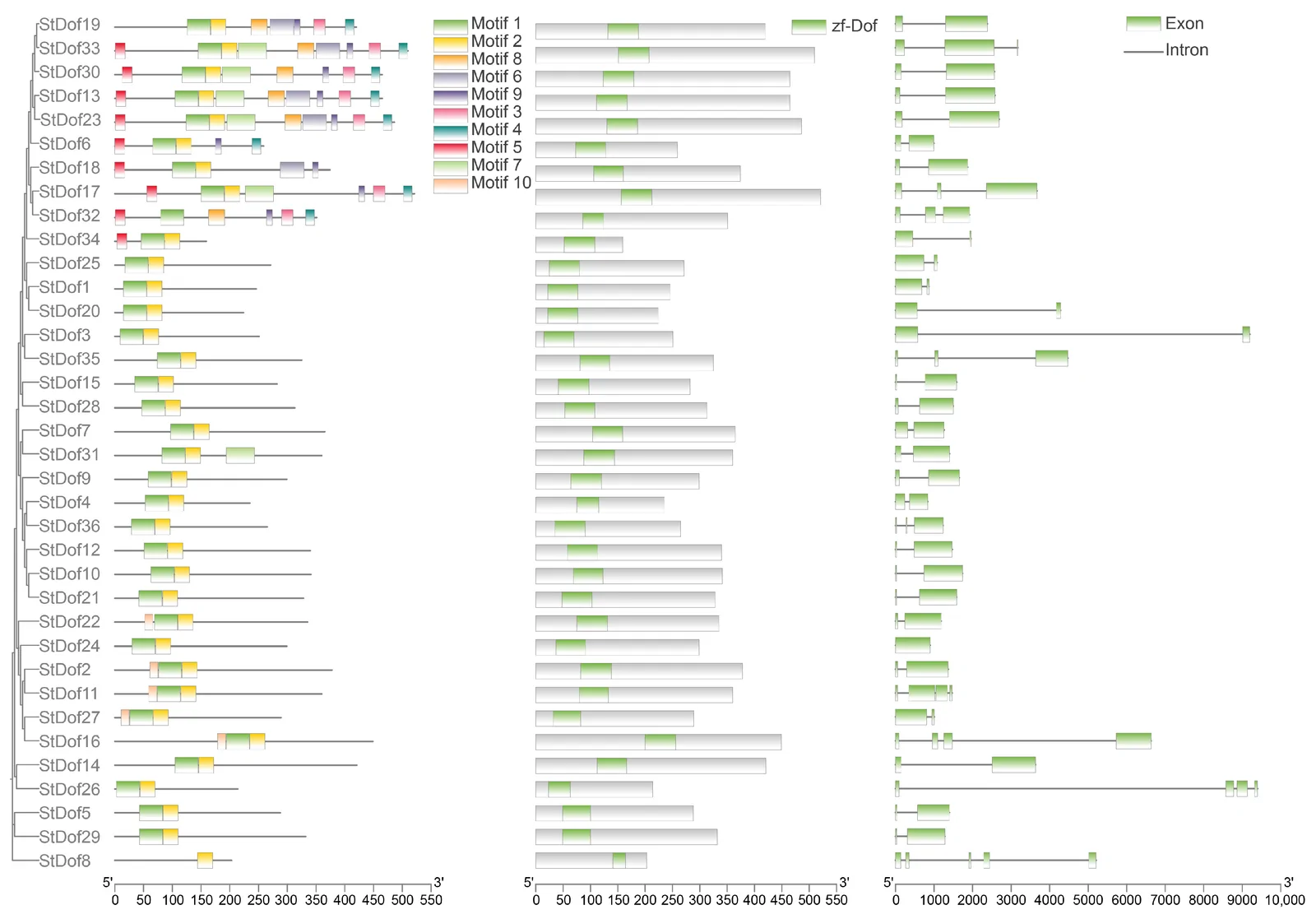
Motif and gene structure analysis of StDof.

To elucidate the structural diversity of *StDof* genes, the exon–intron composition and conserved motifs were systematically analyzed in this study. The results demonstrated significant variation in the number of exons within the *StDof* family (ranging from two to four), with most members containing two exons, suggesting that the gene structure of this family has remained relatively stable throughout evolution (Figure 5).

Ten conserved motifs (Figure 5 and Figure S2) in StDof proteins were identified using the MEME program. Among them, Motifs 1–2 correspond to the conserved Dof domain, which is widely distributed across all subfamilies and constitutes the core functional framework of this family. Conversely, Motifs 3–10 are exclusive to *StDof19/33/30/13/23/6/18/17/32/34*, which are all members of subfamily D. This indicates that subfamily D may have distinct biological functions (Figure 5). In summary, StDof members within the same subfamily exhibit a high degree of consistency in gene structure and motif composition, indicating that they are highly conserved evolutionarily and may perform similar biological functions.

### 3.6. Analysis of Cis-Regulatory Elements in StDof Promoters

**Figure 6:**
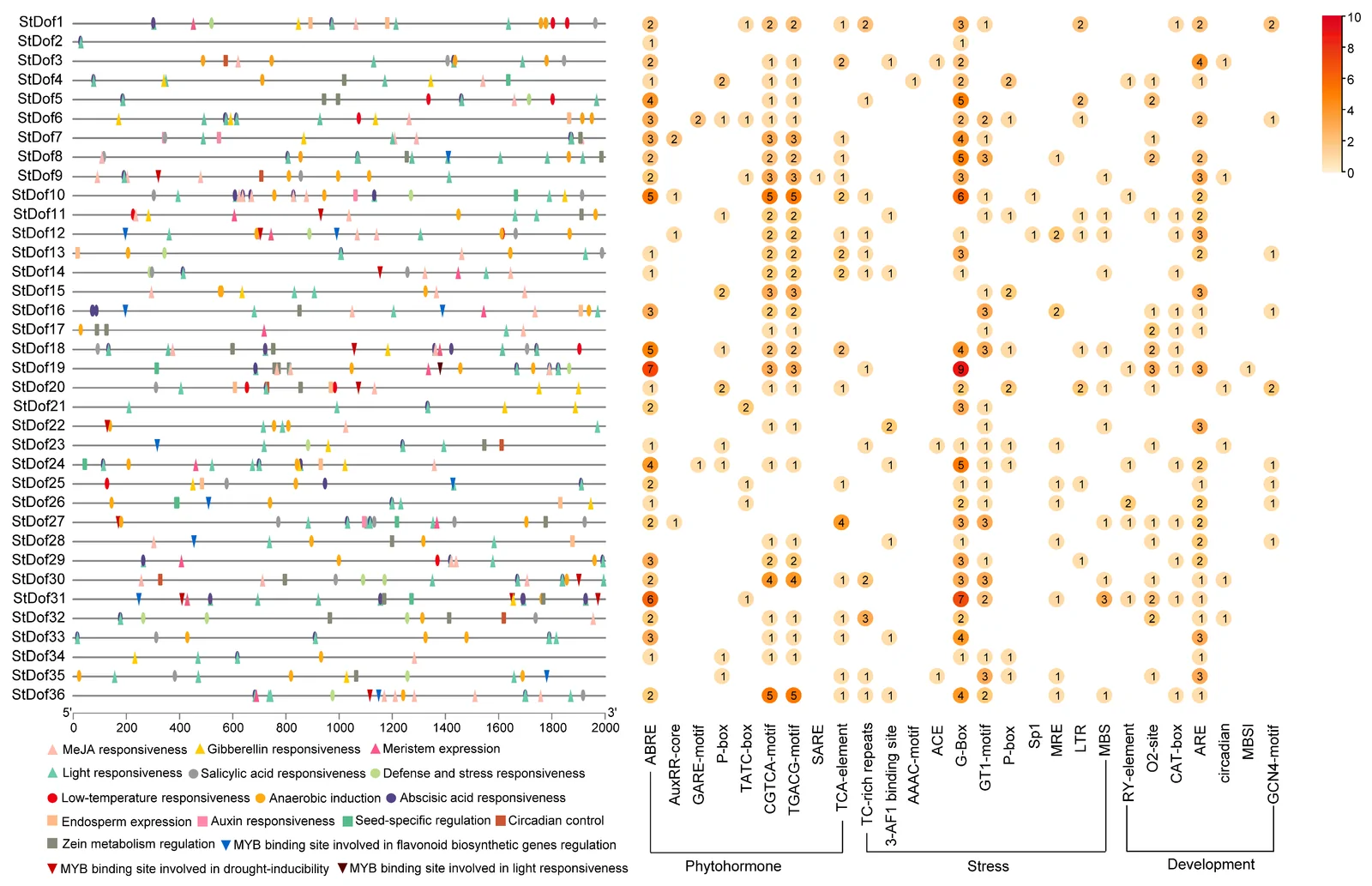
Cis-elements analysis of the *StDof* promoter.

To elucidate the potential functions of *Dof* transcription factors in responses to abiotic or biotic stresses, a systematic analysis of cis-regulatory elements within the 2.0 kb region upstream of the *StDof* promoter was conducted using the PlantCARE database. The results revealed that a total of 26 cis-acting elements were predicted in the *StDof* promoter region, which could be classified into three major categories: development-related elements, hormone-responsive elements, and environmental stress-responsive elements (Figure 6). Development-related elements primarily include anaerobic induction, circadian control, endosperm expression, seed-specific regulation, and zein metabolism regulation. Hormone-responsive elements include abscisic acid responsiveness, auxin responsiveness, gibberellin responsiveness, MeJA responsiveness, and salicylic acid responsiveness, suggesting the potential capacity of this family to participate in hormone-mediated crosstalk between growth and stress tolerance. Among the categories of environmental stress responses, light-responsive elements are the most abundant, comprising at least eight distinct subtypes (Figure 6), which highlights the potential responsiveness of *StDof* family members to light signals. Notably, multiple anaerobic response elements (AREs), as well as cis-regulatory elements such as low-temperature responsiveness (LTR), defense and stress responsiveness (TC-rich repeats), and drought inducibility (MBS), have also been predicted to exist in most *StDof* promoters. In summary, *StDof* genes may not only be involved in fundamental biological processes such as seed development but may also serve as key transcriptional regulatory nodes that integrate various hormonal and environmental signals, implying putative roles in plant response networks to various abiotic stresses, including drought, low temperature, and hypoxia.

### 3.7. Protein–Protein Interaction and miRNA-Target Prediction Analysis of the StDof Gene Family

To investigate the putative potential interaction partners of the StDof proteins in *S. tora*, the amino acid sequences of all 36 StDof members were aligned against the *A. thaliana* protein database to identify homologous proteins, and the corresponding predicted interactions were retrieved from the STRING database. The results indicated that most StDof members may interact with non-Dof proteins such as ADO3, NAC020, NAC028, NAC071, LHW, and AHL4. Notably, ADO3 (also known as FKF1), acting as a blue light receptor and a component of the SCF E3 ubiquitin ligase complex, mediates the degradation of CDF-type Dof proteins via the ubiquitin-proteasome pathway and participates in photoperiod-dependent flowering regulation [30], which suggests that StDof proteins may be regulated by the ubiquitination pathway, although this regulatory mechanism has not been experimentally verified in *S. tora*. Interactions with NAC-like transcription factors suggest that they may potentially be involved in stress responses [31], while interactions with LHW and AHL4 imply that they may potentially be involved in vascular tissue (xylem/proto-meristem) development [32,33]. Furthermore, 12 StDof members have not yet been found to interact with any proteins and are distributed discretely within the network, suggesting that they may possess species-specific regulatory functions.

**Figure 7:**
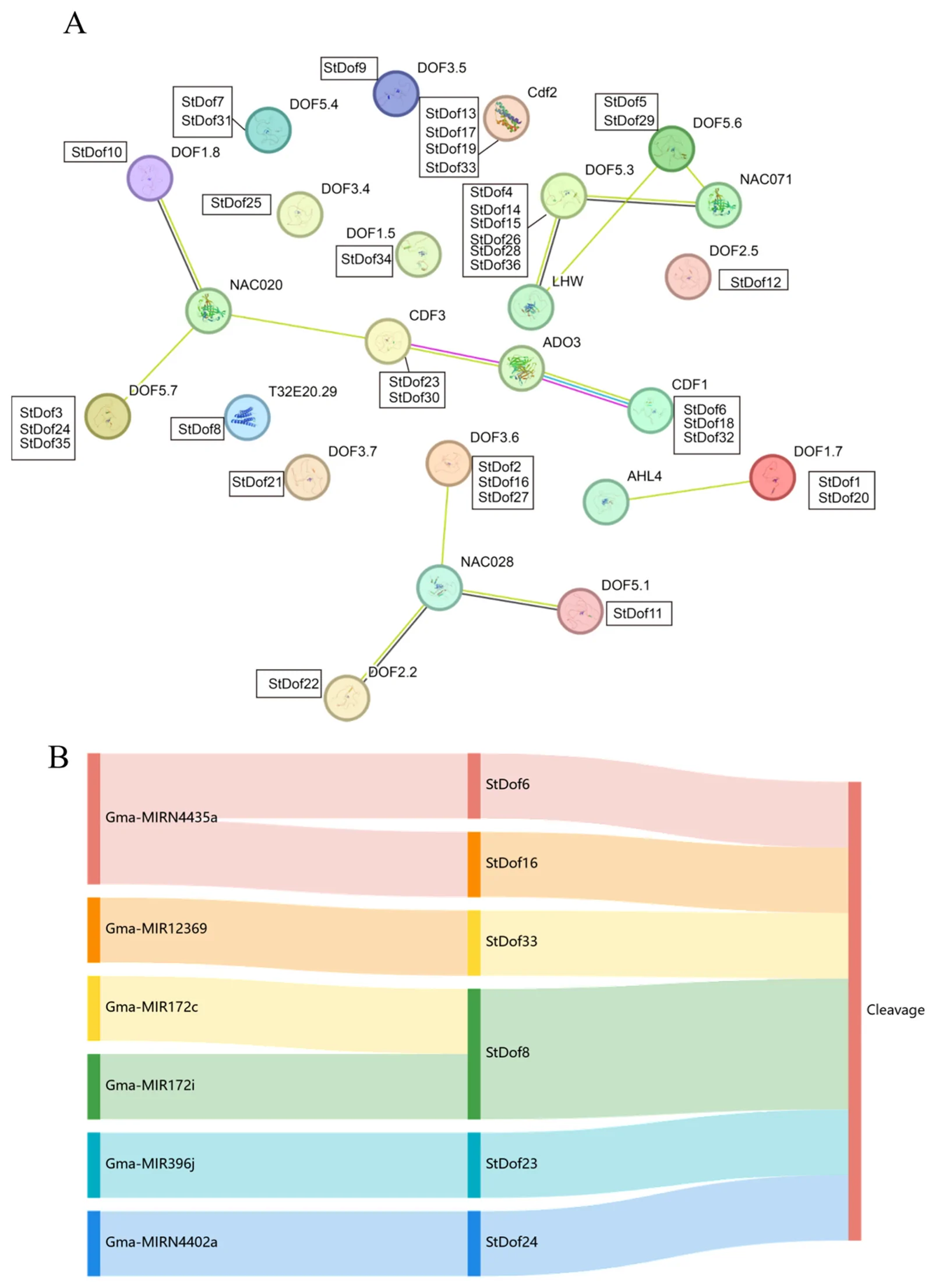
Predicted PPI (**A**) and miRNA–target (**B**) networks of StDof family members. In panel (**B**), each colored ribbon links one miRNA (**left**) to its corresponding target *StDof* gene (**right**). Different ribbon colors are used merely to visually distinguish distinct miRNA-target pairs. The label “Cleavage” on the right denotes the predicted miRNA-mediated cleavage mode for target transcripts.

To further identify miRNAs that may regulate the *StDof* genes in *S. tora*, predictive analysis was performed using psRNATarget. The results (Figure 7B) show that a total of seven high-confidence miRNA–StDof target regulation relationships were identified. Notably, Gma-MIRN4435a was predicted to target two *StDof* genes (*StDof6* and *StDof16)*, indicating that a single miRNA can regulate multiple members within the same family. In contrast, *StDof8* was targeted by two miRNAs (*Gma-MIR172c* and *Gma-MIR172i*), suggesting that an individual *Dof* transcription factor gene can be co-regulated by multiple miRNAs. The remaining *StDof23*, *StDof24*, and *StDof33* are each targeted by a single miRNA. All predicted miRNAs regulate their corresponding *StDof* genes through cleavage.

### 3.8. Expression Patterns of StDof Genes at Different Stages of Seed Development

**Figure 8:**
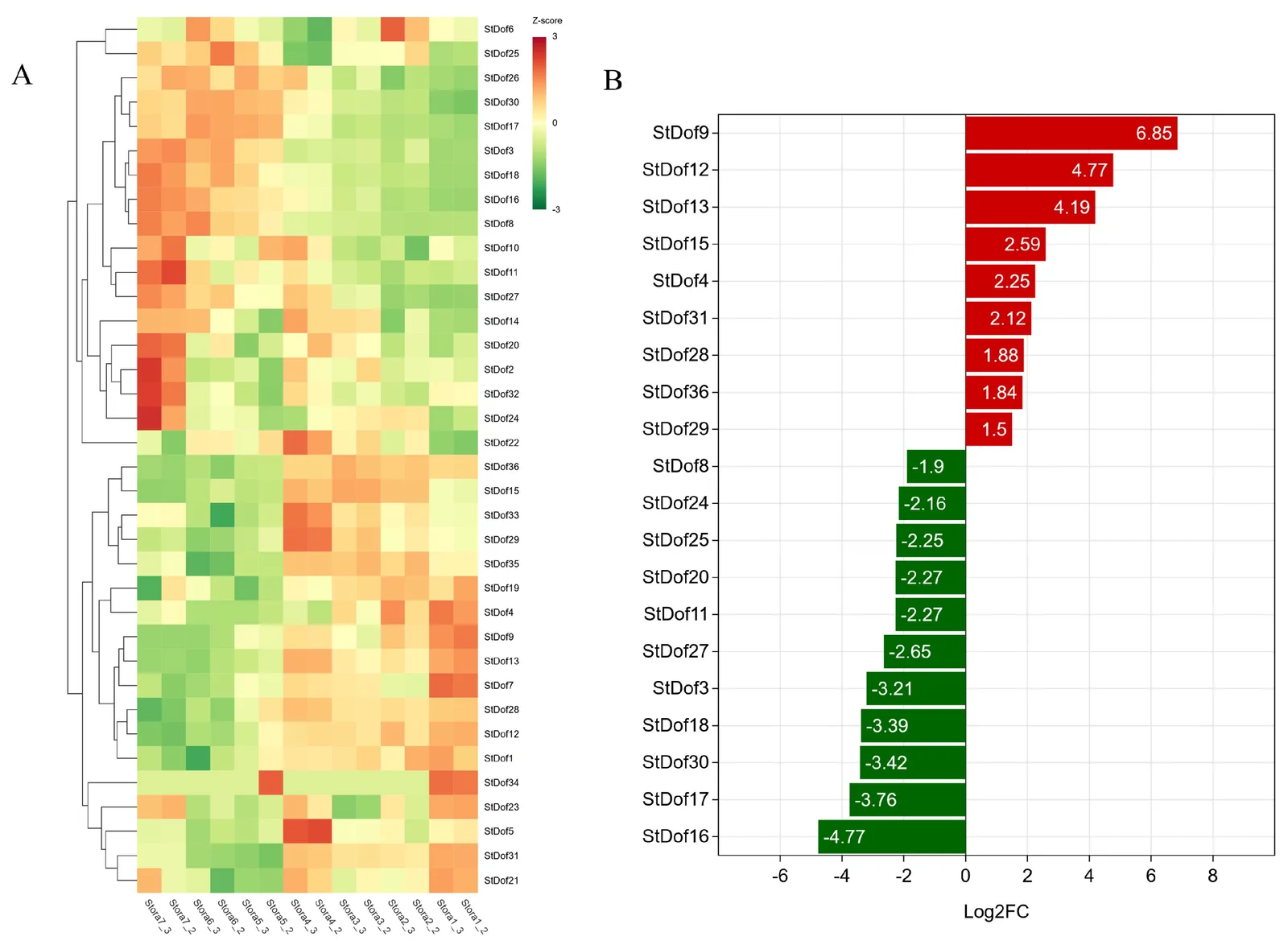
Expression profiling and differential expression analysis of *StDof* genes. (**A**) Expression profile of *StDof* genes at different stages of seed development. (**B**) Differential expression analysis of *StDof* genes. The *x*-axis represents log_2_FC, and the *y*-axis indicates gene names. DEGs were identified using the threshold of |log_2_FC| ≥ 1.5 and *p* < 0.05.

To explore the expression patterns of *StDof* genes at different stages of seed development, a clustered heatmap was constructed based on RNA-seq data downloaded from the NCBI database. The expression profile results demonstrated that the 36 *StDof* genes were grouped into two major clusters. In group A, 18 *StDof* genes were prominently expressed during the first four stages of seed development and weakly expressed during the last three stages, whereas the remaining 18 genes were prominently expressed during the last three stages and weakly expressed during the first four stages (Figure 8A). Further differential expression analysis of transcriptomic data from the early and late stages of seed development revealed that *StDof9* showed the greatest upregulation, while *StDof16* exhibited the most significant downregulation (Figure 8B), suggesting that *StDof9* and *StDof16* have a potential antagonistic functional relationship during the development of *S. tora* seeds.

**Figure 9:**
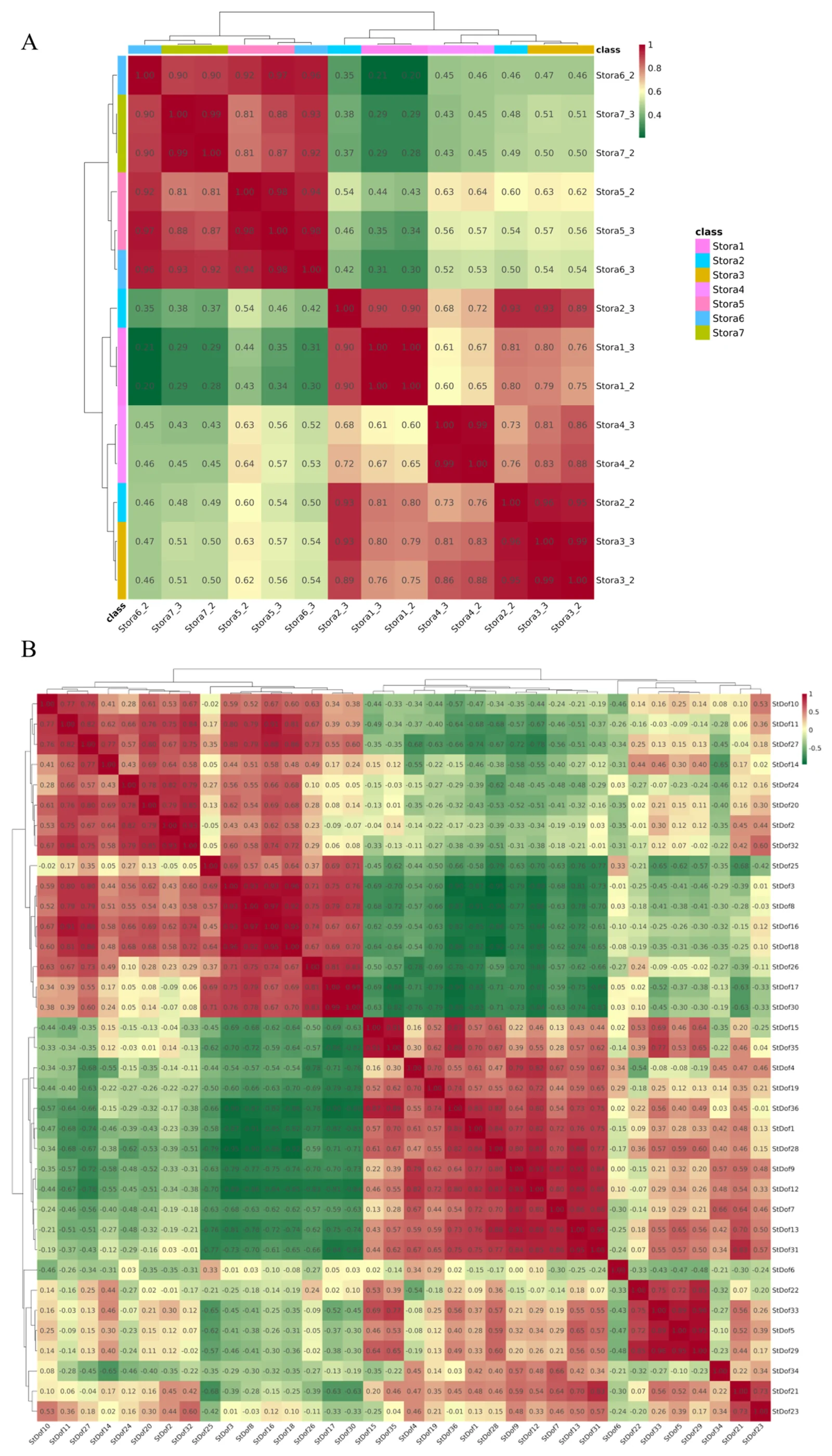
Relationship between the samples (**A**) and *StDof* gene expression (**B**) at different stages of seed development. Pearson correlation was used to assess the correlation between gene pairs and between samples.

Subsequently, the correlations of *StDof* gene expression patterns among samples from different seed developmental stages were analyzed. The results showed that the overall expression similarity among samples from the seven seed developmental stages exhibited a decreasing trend as development progressed. Specifically, high expression profile correlations were maintained among samples from the first four early developmental stages and among those from the last three late developmental stages, whereas the correlation between samples from the early and late developmental stages decreased markedly (Figure 9A). These findings suggest that the expression patterns of *StDof* genes in *S. tora* are not stochastic fluctuations but rather exhibit clear developmental stage specificity. These genes may retain relatively conserved co-expression features during both early and late seed development, accompanied by putative transcriptional reprogramming at critical transitional nodes of the developmental process.

Furthermore, these *StDof* genes were divided into two groups according to their correlation patterns across seed-development stages (Figure 9B). One group showed predominantly significant negative correlations, including *StDof10*, *StDof11*, *StDof27*, *StDof14*, *StDof24*, *StDof20*, *StDof2*, *StDof32*, *StDof25*, *StDof3*, *StDof8*, *StDof18*, *StDof16*, *StDof26*, *StDof17*, and *StDof30*. The other group exhibited predominantly significant positive correlations, consisting of *StDof15*, *StDof35*, *StDof4*, *StDof19*, *StDof36*, *StDof1*, *StDof28*, *StDof9*, *StDof12*, *StDof7*, *StDof13*, *StDof31*, *StDof6*, *StDof22*, *StDof33*, *StDof5*, *StDof29*, *StDof34*, *StDof21*, and *StDof23*. These contrasting correlation patterns suggest that the expression of *StDofs* at different seed developmental stages might be associated with opposing expression trends among different *StDof* members.

### 3.9. Subcellular Localization of StDof9 and StDof16

**Figure 10:**
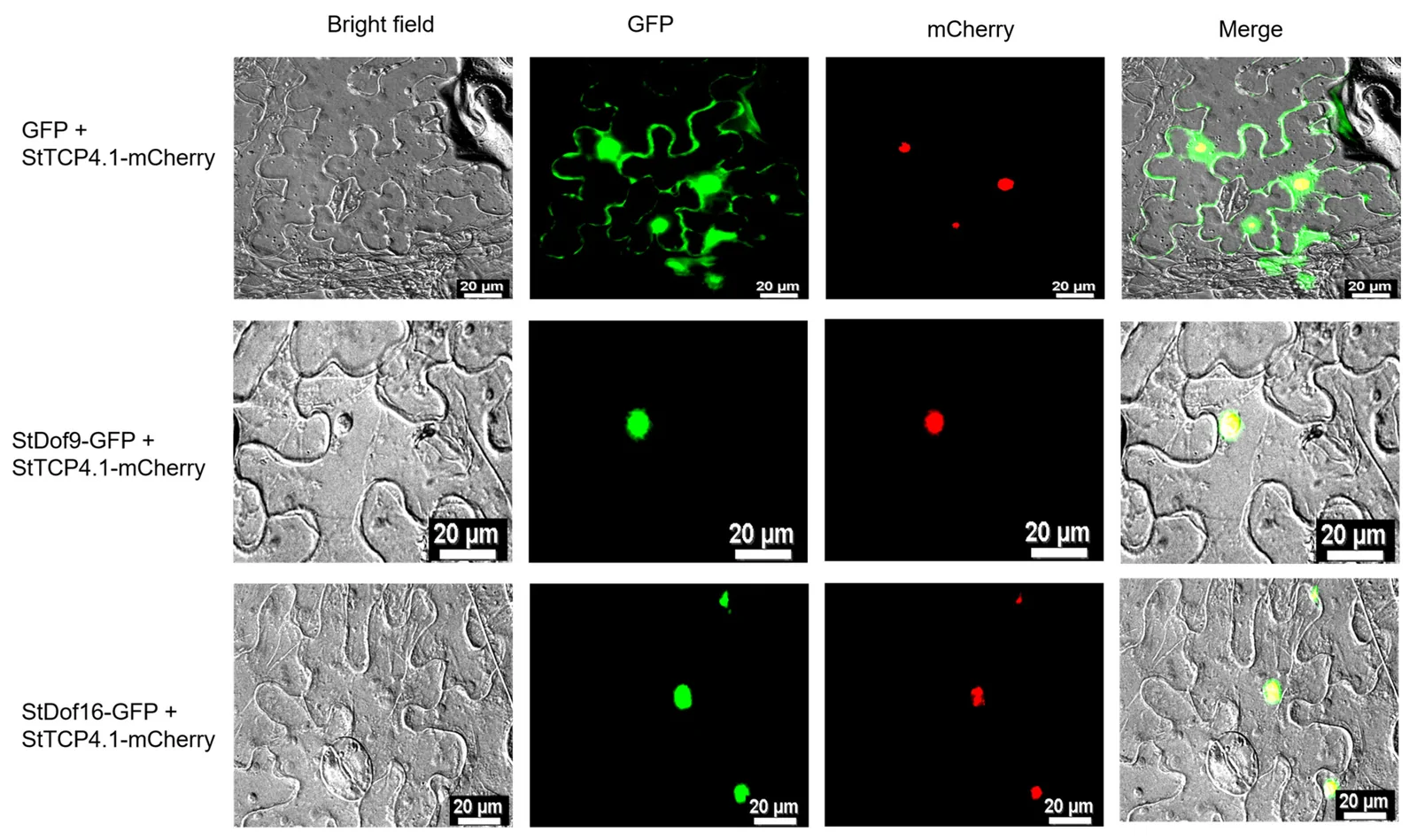
Subcellular localization of *StDof9* and *StDof16*. Scale bar = 20 μm.

Since the seeds of *S. tora* constitute its medicinal part, seed development is a direct determinant of both yield and quality. Previous results showed that *StDof9* was upregulated to the greatest extent during seed development, while *StDof16* was the most markedly downregulated (Figure 8B), implying that these two genes may function antagonistically in this process. To determine the subcellular distribution patterns of *StDof9* and *StDof16*, the complete coding sequences of both genes were amplified and used to construct 35S promoter-driven StDof9-GFP and StDof16-GFP fusion expression vectors, which were subsequently transformed into tobacco leaves for transient expression. Subcellular fluorescence observation revealed that the GFP signal of the empty vector (EV) was diffusely distributed across various organelles in tobacco cells, whereas the fluorescence of StDof9-GFP and StDof16-GFP fusion proteins was specifically restricted to the nucleus (Figure 10), suggesting that *StDof9* and *StDof16* primarily exert their biological functions within the nucleus.

## 4. Discussion

In recent years, with the significant reduction in sequencing costs and marked improvement in genome assembly quality, genome-wide identification of *Dof* transcription factors has been extensively conducted in various plant species, and the number of family members varies significantly among different species. Among model plants, *A. thaliana* and rice contain 36 and 30 *Dof* genes, respectively [7], whereas in soybean, which has undergone multiple whole-genome duplication (WGD) events, the family has expanded markedly to 79 members [34]. Studies on non-model plants have further corroborated this diversity, with 51, 45, and 28 *Dof* genes identified in the genomes of blueberry, *Camellia oleifera* (*C. oleifera*), and *Eugenia uniflora* (*E. uniflora*), respectively [35,36,37], while *S. tora* contains 36 members. These data indicate that the *Dof* gene family has undergone complex lineage-specific expansions or contractions across different plant lineages.

Phylogenetic results indicate that StDof proteins are classified into four classical subfamilies (A, B, C, and D) and further subdivided into nine subgroups (A, B1, B2, C1, C2.1, C2.2, C3, D1, and D2), which is consistent with the classification framework established for AtDof proteins and subsequently validated across multiple species [38]. Subgroup C contained 13 StDof proteins (the largest proportion), whereas subgroup A contained only 6 (the smallest proportion), consistent with the pattern observed in multiple angiosperm lineages. In *Dendrobium officinale* (*D. officinale*), the D subgroup is dominant, while the A subgroup has the fewest members [39]. Similarly, in *Medicago polymorpha* (*M. polymorpha*), the Dof family also exhibits a distribution characterized by an abundance of Subgroup D members and a scarcity of Subgroup A members [40]. Furthermore, Dof members of subgroup D are generally involved in the regulation of carbon metabolism and seed storage proteins. Given that the major bioactive constituents of *S. tora* are predominantly enriched in seeds, it is speculated that the 13 StDof members identified in subgroup D may play critical roles in seed development and secondary metabolite accumulation in *S. tora*.

All 36 StDof proteins contain a conserved C2-C2-type zinc finger DNA-binding domain, the hallmark of the Dof family, which recognizes the AAAG core motif in target gene promoters [41]. This domain is universally present in all identified members, suggesting that the core DNA-binding function has been conserved throughout the evolution of StDof, while regulatory specificity may be achieved through the diversification of flanking motifs and protein-interaction surfaces.

Collinearity analysis of the *StDof* gene family provides a detailed portrait of the evolutionary forces that have shaped their expansion. A total of 23 collinear gene pairs were identified in the *S. tora* genome. The Ka/Ks ratios for all collinear pairs were <1 (0–0.51), confirming that purifying selection has been the dominant evolutionary pressure acting on duplicated *StDof* genes. This result is consistent with the functional essentiality of *Dof* transcription factors, as loss-of-function mutations in *Dof* genes frequently produce severe phenotypes, including impaired seed germination in *A. thaliana* and defective starch accumulation in maize [15,42], suggesting that strong selective constraints act to preserve coding sequence integrity.

The cross-species collinearity analysis revealed that the *Dof* genes in *S. tora* share 54 pairs of orthologs with *A. thaliana*, compared to only 16 pairs with rice, reflecting the closer phylogenetic relationship between *S. tora* and *A. thaliana*, both of which are dicotyledons. Furthermore, *StDof27* and *StDof30* exhibited five colinear relationships with *A. thaliana Dof* genes, respectively, while *StDof27* exhibited four colinear relationships with rice. This “one-to-many” collinearity is a hallmark of gene families amplified by species-specific duplication events [11]. Therefore, the functional characterization of these *StDof* members with multiple colinear relationships may reveal their functional roles in *S. tora*-specific biological processes.

The protein–protein interaction (PPI) network of StDof proteins in this study was constructed by mapping StDof sequences to their *A. thaliana* orthologs and retrieving interaction information from the STRING database. Therefore, these interactions were inferred from orthologous proteins in *A. thaliana*, rather than experimentally validated protein interactions in *S. tora*. Protein interaction patterns may differ between species due to divergence in protein sequences and regulatory networks during evolution. Thus, the predicted PPI relationships should be interpreted cautiously, and further in vitro or in vivo experiments, such as yeast two-hybrid and bimolecular fluorescence complementation assays, are required to verify these potential interactions in *S. tora*.

*Dof* transcription factors play a key role in the completion of seed storage compound accumulation, drought tolerance, and dormancy. In *A. thaliana*, studies have shown that the *Dof* transcription factor DOF6 forms a complex with the DELLA protein RGL2 and regulates primary seed dormancy by transcriptionally regulating GATA [42]. Similarly, in maize, *ZmDOF36* activates starch biosynthesis genes, including *ZmGBSSI* and *ZmISA1*, during endosperm development [15]. In rice, multiple *Dof* genes exhibit endosperm-preferential expression patterns during seed filling [43]. Transcriptomic analysis of *StDof* genes across seven seed developmental stages in *S. tora* revealed that 18 genes were predominantly expressed during the first four stages (early development), whereas the remaining 18 genes were predominantly expressed during the last three stages (late development). Therefore, *StDof* genes are speculated to be potentially correlated with the biosynthesis of storage proteins, drought tolerance, and seed dormancy.

Transient expression of a GFP fusion protein in tobacco leaves confirmed that *StDof9* and *StDof16* were localized to the cell nucleus. Nuclear localization is a universal property of Dof proteins and has been experimentally validated for numerous members, including *ZmDOF36* in maize [15], *DoDof15/22/24* in *D. officinale* [39], and *CmDOF18* in chrysanthemum [44]. In contrast to the absence of GFP signal in other organelles, this result confirms that the nuclear localization of *StDof9* and *StDof16* is specific and mediated by functional nuclear localization signals in their protein sequences.

In medicinal plants, 48 *Dof* members have been identified in *Prunella vulgaris* (*P. vulgaris*) and 28 *Dof* genes in *D. officinale*, whereas 36 *StDof* genes were identified in *S. tora* in this study. The Dof proteins from all three species contain a typical C2-C2-type zinc-finger Dof domain, which is responsible for DNA binding and is a signature conserved feature of the *Dof* transcription factor family. Analysis of promoter cis-acting elements in all three studies identified a large number of elements associated with light response, hormone response, and growth and development [2,39]. Notably, the transcriptomic data of *S. tora* showed that *StDof* genes exhibit a distinct stage-preferential expression pattern during seed development. Given that *S. tora* is used medicinally in the form of seeds, this expression pattern suggests that the *StDof* genes may play a potential regulatory role in seed growth and development. It should be noted that the transcriptomic data analyzed in this study were retrieved from public databases, and original experimental materials were unavailable. Owing to the long growth cycle of *S. tora*, qRT-PCR validation across seed developmental stages and heterologous functional assays could not be performed in the current work. Therefore, all regulatory inferences regarding *StDof* genes are based on bioinformatic analyses, transcriptome profiles, and subcellular localization results. Its biological function in *S. tora* still requires further experimental evidence to confirm.

## 5. Conclusions

In summary, this study systematically identified and analyzed the *StDof* gene family in *S. tora*, elucidating the evolutionary history, structural conservation, cis-regulatory potential, and dynamic expression patterns of this gene family during seed development. It also confirmed that *StDof9* and *StDof16* exert biological functions within the nucleus. The results of this study lay the theoretical foundation for subsequent functional validation experiments on pivotal *StDof* genes and will help advance molecular breeding research aimed at increasing *S. tora* seed yield and enriching its medicinal active components.

## Figures and Tables

**Table 1:** Overview of *Dof* gene families in *S. tora*.

Gene ID	Gene Name	Number of Amino Acids (aa)	Molecular Weight (kDa)	Theoretical pI	Hydropathicity	Prediction of Subcellular Localization
gene-G2W53_000362	StDof1	246	26.35	8.84	−0.835	Nucleus
gene-G2W53_002415	StDof2	378	39.39	8.99	−0.455	Nucleus
gene-G2W53_002583	StDof3	251	28.17	7.68	−0.631	Chloroplast
gene-G2W53_002584	StDof4	235	25.38	7.80	−0.185	Plasma membrane
gene-G2W53_006810	StDof5	288	32.27	6.04	−0.803	Nucleus
gene-G2W53_006953	StDof6	259	29.58	8.61	−0.977	Chloroplast
gene-G2W53_007042	StDof7	365	40.18	6.89	−0.550	Nucleus
gene-G2W53_012216	StDof8	203	22.23	9.06	0.238	Mitochondrion
gene-G2W53_012982	StDof9	299	33.50	5.86	−0.663	Nucleus
gene-G2W53_013079	StDof10	341	36.34	9.21	−0.813	Nucleus
gene-G2W53_016848	StDof11	360	37.06	9.48	−0.470	Nucleus
gene-G2W53_017371	StDof12	340	37.14	8.83	−0.734	Nucleus
gene-G2W53_019206	StDof13	465	50.95	6.57	−0.827	Nucleus
gene-G2W53_019929	StDof14	421	44.94	9.01	−0.370	Nucleus
gene-G2W53_021596	StDof15	282	32.03	6.96	−1.023	Nucleus
gene-G2W53_021757	StDof16	449	49.52	9.01	−0.767	Nucleus
gene-G2W53_022960	StDof17	521	57.65	8.80	−0.618	Nucleus
gene-G2W53_024690	StDof18	374	40.83	7.52	−0.755	Nucleus
gene-G2W53_025976	StDof19	420	46.04	5.73	−0.901	Nucleus
gene-G2W53_028138	StDof20	224	23.55	9.32	−0.529	Nucleus
gene-G2W53_030636	StDof21	328	35.42	8.50	−0.802	Nucleus
gene-G2W53_032306	StDof22	335	36.52	9.12	−0.524	Nucleus
gene-G2W53_035855	StDof23	486	53.02	8.55	−0.789	Nucleus
gene-G2W53_036149	StDof24	299	31.79	8.50	−0.583	Nucleus
gene-G2W53_036529	StDof25	271	28.01	6.53	−0.407	Cell wall
gene-G2W53_036537	StDof26	214	22.76	8.66	−0.668	Nucleus
gene-G2W53_037631	StDof27	289	31.45	9.53	−0.506	Nucleus
gene-G2W53_037712	StDof28	313	34.60	7.44	−0.734	Nucleus
gene-G2W53_038244	StDof29	332	36.62	6.29	−0.844	Nucleus
gene-G2W53_038365	StDof30	465	50.29	8.37	−0.641	Nucleus
gene-G2W53_038450	StDof31	360	38.84	6.51	−0.630	Nucleus
gene-G2W53_040535	StDof32	351	38.46	6.20	−0.684	Nucleus
gene-G2W53_042106	StDof33	510	55.37	5.86	−0.848	Nucleus
gene-G2W53_042440	StDof34	159	18.07	9.34	−0.998	Chloroplast
gene-G2W53_042541	StDof35	325	35.42	8.56	−0.694	Nucleus
gene-G2W53_042542	StDof36	265	28.87	7.07	−0.903	Nucleus

## Data Availability

The genomic (No. ASM1485142v1) and transcriptome data from different stages of seed development (SRX7700171-SRX7700176 and SRX7700182-SRX7700189) were retrieved from the NCBI SRA database (http://www.ncbi.nlm.nih.gov/sra, accessed on 12 July 2026). All analytical results generated in this work are provided within the article and Supplementary Materials.

## Supplementary Materials

The following supporting information can be downloaded at: https://www.mdpi.com/article/10.3390/biology15201832/s1, Figure S1. Sequence alignment of Dof proteins. Figure S2. Motifs of StDof proteins. Table S1. Primers used in this study. Table S2. Secondary structure analysis of Dof proteins in *S. tora.* Table S3. Ka, Ks, and Ka/Ks values of Dof proteins in *S. tora*.
