# Supplementary material for: Genome-Wide Dissection of the Dof Transcription Factor Family in Senna tora: From Gene Identification to Expression Signatures
Source: Biology (Basel). 2026 Oct 13;15(20):1832. doi: 10.3390/biology15201832 (PMC13701675; doi:10.3390/biology15201832)
Supplement: Supplementary file 1 [file biology-15-01832-s001.zip › biology-4511465-supplementary.pdf]

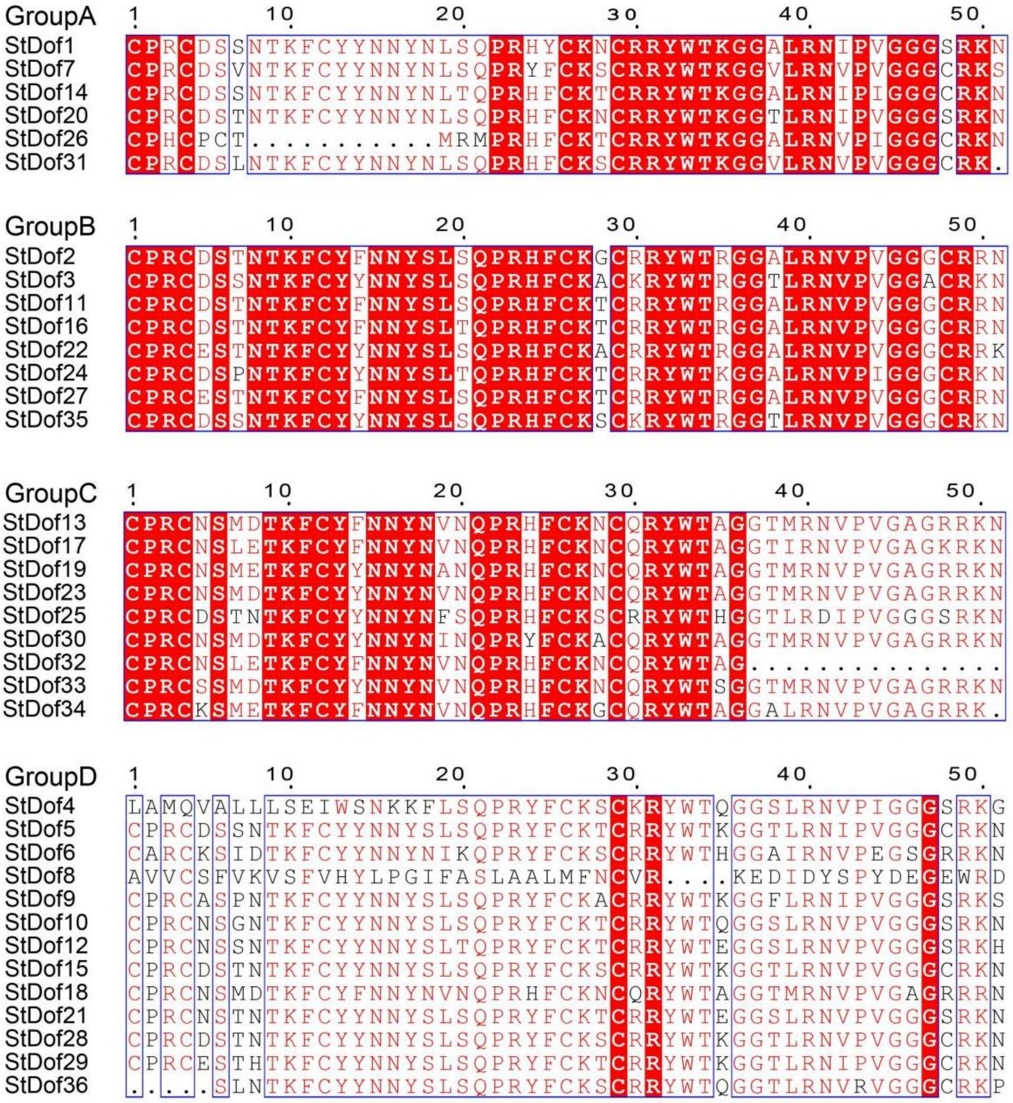

Supplemental Figure S1. Sequence alignment of Dof proteins.

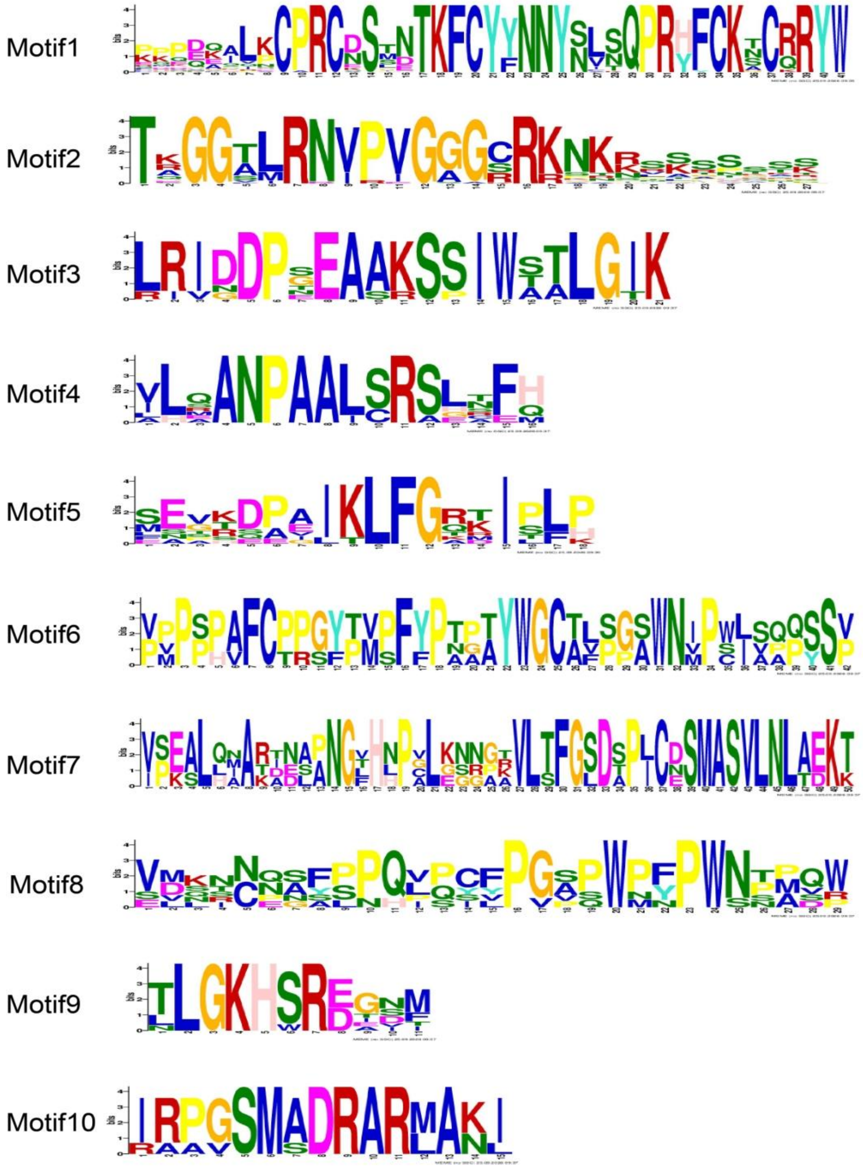

Supplemental Figure S2. Motifs of StDof proteins.

Supplemental Table S1. Primers used in this study.

Primer Names	Primer sequences (5'-3')
StDof9-GFP-F	atttacgaacgatagccatggTGATGTTCAAGCATAGGAGACCACG
StDof9-GFP-R	cctcgcccttgctcaccatggCATGAGAAGAATGGAGAGTGGAAAAG
StDof16-GFP-F	atttacgaacgatagccatggTGATGCGAGAAAGCACTCATAATTTC
StDof16-GFP-R	cctcgcccttgctcaccatggCCAAGACATGGCTAGTGGAAGAGAA

Supplemental Table S2. Secondary structure analysis of Dof proteins in *S. tora*

Protein	Proportion of α-helix	Proportion of extended strand	Proportion of β-turn	Proportion of random coil
StDof1	6.10	4.88	0.81	88.21
StDof2	5.29	5.03	0.53	89.15
StDof3	7.57	4.78	0.80	86.85
StDof4	4.26	8.51	0.85	86.38
StDof5	3.82	8.33	1.39	86.46
StDof6	11.97	8.88	1.54	77.61
StDof7	7.67	4.11	1.37	86.85
StDof8	27.09	17.24	4.93	50.74
StDof9	7.02	5.35	1.00	86.62
StDof10	2.35	6.74	0.59	90.32
StDof11	6.94	3.89	0.83	88.33
StDof12	4.71	5.59	0.59	89.12
StDof13	6.67	7.31	0.86	85.16
StDof14	14.49	2.38	0.95	82.19
StDof15	7.45	4.96	0.71	86.88
StDof16	16.48	7.57	3.12	72.83
StDof17	5.76	6.33	0.58	87.33
StDof18	2.14	5.88	1.34	90.64
StDof19	6.43	6.67	0.48	86.43
StDof20	12.05	4.46	1.79	81.70
StDof21	3.05	6.71	0.61	89.63
StDof22	5.97	6.57	0.60	86.87
StDof23	7.41	6.79	1.03	84.77
StDof24	4.01	4.01	0.67	91.30
StDof25	5.17	9.23	1.48	84.13
StDof26	5.14	1.87	0.93	92.06
StDof27	4.15	7.96	2.08	85.81
StDof28	7.03	7.03	0.64	85.30
StDof29	3.01	4.22	0.60	92.17
StDof30	7.31	5.81	0.65	86.24
StDof31	4.17	5.00	1.11	89.72
StDof32	9.40	7.69	0.57	82.34
StDof33	5.88	6.47	0.78	86.86
StDof34	3.77	10.06	1.26	84.91
StDof35	5.23	5.54	0.62	88.62
StDof36	5.66	4.53	1.51	88.30

Supplemental Table S3. Ka, Ks, and Ka/Ks values of Dof proteins in *S. tora*

Gene 1	Gene2	Ka	Ks	Ka/Ks
StDof1	StDof20	0.25306426322754355	0.839657464020096	0.30138988107832365
StDof2	StDof11	0.13965845762533396	0.9434535529569359	0.14802896993457895
StDof4	StDof36	0.553512014328763	1.9242960165683347	0.2876439017505532
StDof5	StDof29	0.3288602945326501	1.0915171111647757	0.30128734691270015
StDof6	StDof13	0.44119616631951714	4.4208021256983585	0.09980002582672051
StDof6	StDof23	0.4418087919299293	NaN	NaN
StDof6	StDof30	0.29979458040936724	0.7632367576317699	0.39279368742615733
StDof7	StDof31	0.30682081044661597	0.7391127452257912	0.41512044329973696
StDof10	StDof12	0.46413767759189845	0.922646999568776	0.5030501132164584
StDof10	StDof21	0.3189291198692862	1.0171384197363873	0.31355527790597404
StDof11	StDof16	0.4408399986765892	1.4343660113356407	0.30734135861605616
StDof12	StDof21	0.4327397912458255	1.3460655929043452	0.32148492133442197
StDof13	StDof23	0.1623178391619568	0.7028107114088531	0.23095527220490814
StDof13	StDof30	0.2904269133673244	2.166910260131651	0.13402812230428013
StDof13	StDof33	0.3494981414086952	2.2034367628648592	0.15861500874401566
StDof14	StDof26	0.5105629358863323	1.0217503679970545	0.4996943988258041
StDof15	StDof28	0.25721020525363336	1.000356125885199	0.25711863865084267
StDof16	StDof27	0.2729538273679254	1.1003061904680722	0.24807079132383172
StDof18	StDof32	0.24917998941884167	1.1677789938855156	0.21337940716826276
StDof19	StDof33	0.19845722213119316	0.6843911724371354	0.2899763032075379
StDof22	StDof27	0.5655505157413393	2.234193866428196	0.2531340382943063

StDof23	StDof30	0.3361155016678228	2.40661911676103	0.1396629401499091
StDof23	StDof33	0.3331057387354948	2.0509296124584044	0.16241695312800533
